# Hashimoto’s Thyroiditis, microcalcification and raised thyrotropin levels within normal range are associated with thyroid cancer

**DOI:** 10.1186/1477-7819-11-56

**Published:** 2013-03-05

**Authors:** Zhi-qiang Ye, Dian-na Gu, Hong-ye Hu, Yi-li Zhou, Xiao-qu Hu, Xiao-hua Zhang

**Affiliations:** 1 Department of Surgical Oncology, The First Affiliated Hospital of Wenzhou Medical College, Wenzhou 325000, China; 2 Department of Infection and Liver Diseases, The First Affiliated Hospital of Wenzhou Medical College, Wenzhou 325000, China

**Keywords:** Thyroid cancer, Thyroiditis, Papillary thyroid cancer, Thyrotropin

## Abstract

**Background:**

To confirm whether clinical and biochemical parameters or Hashimoto’s thyroiditis (HT) could predict the risks of malignancy among subjects who underwent thyroidectomy, as well as to determine the influence of HT on the biological behavior of papillary thyroid cancer (PTC).

**Methods:**

A total of 2,052 patients who underwent initial thyroidectomy were enrolled between June 2006 and August 2008. Serum free T4, free T3, thyrotropin (TSH), thyroglobulin, thyroglobulin antibody, antimicrosomal antibody, tumor-associated status, and thyroid disorders were documented.

**Results:**

Binary logistic regression analysis was performed to define the risk predictors for thyroid cancer. Finally, calcification, HT, TSH, and age, were entered into the multivariate model. Multivariate logistic regression analysis revealed the risk of thyroid cancer increases in parallel with TSH concentration within normal range, and the risk for malignancy significantly increased with serum TSH 1.97–4.94 mIU/L, compared with TSH less than 0.35 mIU/L (OR = 1.951, 95% CI = 1.201–3.171, *P* = 0.007). Increased risks of thyroid cancer were also detected among the patients with HT (OR = 3.732, 95% CI = 2.563–5.435), and microcalcification (OR = 14.486, 95% CI = 11.374–18.449). The effects of HT on the aggressiveness of PTC were not observed in extrathyroidal invasion (*P* = 0.347), capsular infiltration (*P* = 0.345), angioinvasion (*P* = 0.512), and lymph node metastases (*P* = 0.634).

**Conclusions:**

The risk of malignancy increases in patients with higher level TSH within normal range, as well as the presence of HT and microcalcification. No evidence suggests that coexistent HT alleviates the aggressiveness of PTC.

## Background

Thyroid cancer is the most frequent endocrine malignancy, accounting for approximately 1% of all malignant tumors in the United States [1]. However, a recent study [2] by the Office of Cancer Prevention and Treatment indicates that thyroid cancer represents 5.90% of all new malignant diseases in one district of Wenzhou (a coastal city in Southeast China, with a referral population of 7,558,000). The detection rate of thyroid nodules is higher than before because of high-resolution ultrasound [3]. In order to identify patients with thyroid cancer who require early intervention, many predisposing factors for malignancy have been recognized, including young age (<20 years old) or older age (>70 years old), male gender, large (>4 cm) or rapidly growing nodules, and history of radiation exposure, calcification and thyroglobulin (Tg) [4-7]. The role of thyroid-stimulating hormone (TSH) in the growth and development of thyroid cancer has long been known [8]. Recently, Boelaert *et al*. [9] proposed that the TSH level at presentation is a novel predictor of malignancy in patients with thyroid nodules. Another report indicated that higher TSH levels were associated with greater risks of differentiated thyroid cancer and advanced tumor stage [10]. The findings above need to be verified further.

Hashimoto’s thyroiditis (HT) is an autoimmune disease characterized by widespread lymphocyte infiltration, fibrosis, and parenchymal atrophy [11]. Historically, HT was thought to be associated with malignant lymphoma. However, Dailey *et al*. in 1955 first reported an increased association between HT and papillary thyroid cancer (PTC) [12]. The coexistence of these two diseases has been variously reported to range from 0.5% to 38.0% [11,13-18]. A few studies have proposed HT as a risk factor for thyroid cancer [12,16,18,19], whereas others have reported a negative correlation between the two diseases [10,13,15]. Nowadays, the causal association between these diseases remains controversial, and few papers have discussed whether HT influences the clinical and pathologic features of thyroid cancer.

Therefore, the current study investigates which clinical features or biochemical criteria predict the likelihood of thyroid malignancy in patients with thyroid nodules, thereby identifying those at greatest risk of harboring thyroid cancer. Other objectives of the current study are to determine the following: 1) the relative incidence of HT and PTC among patients undergoing thyroidectomy, and 2) the possible influence of HT on the clinical behavior and neoplastic aggressiveness of PTC.

## Methods

A total of 2,052 patients (1,645 female and 407 male) with thyroid nodules, in whom malignancy was suspected under fine-needle aspiration (FNA) or ultrasonography, or who had rapidly growing nodules or nodules fixed to adjacent structures, and who underwent initial thyroidectomy in the First Affiliated Hospital of Wenzhou Medical College, were retrospectively recruited from June 2006 to August 2008. However, some female patients underwent surgery because of cosmetic problems or anxiety about the effects on their quality of life. The majority of the patients received treatment for thyroid cancer according to the accepted protocol, which involves total or near-total thyroidectomy, followed by thyroid hormone suppression. Cervical lymph node dissection or sentinel lymph node biopsy was performed routinely during thyroid cancer’s operation. Others underwent resection of the whole or partial lobes of the thyroid gland.

The diagnosis was confirmed either on reevaluation of histopathology sections when available, or on reviewing the previous pathology reports. The histologic type of thyroid cancer was assessed by two senior pathologists according to the World Health Organization criteria and classified as PTC in 1004 patients (98.05%), including two with coexisting PTC and malignant lymphoma, follicular thyroid cancer (FTC) in four patients (0.39%), medullary thyroid cancer in twelve patients (1.17%), and anaplastic thyroid cancer in four patients (0.39%). Patients who were characterized by the presence of diffuse lymphocytic and plasma cell infiltration, oxyphilic cells, and lymphoid follicles with reactive germinal centers, were defined as having HT. Whereas patients with lymphocytic infiltration immediately surrounding the malignant tumor (but without other lobe involvement) were considered as having thyroid cancer alone because the surrounding inflammation may represent an immune response to the host tumor [20].

In addition to the demographic data (age and gender), data on the thyroid-associated parameters [serum free T4 (fT4), free T3 (fT3), TSH, Tg, and thyroglobulin antibody (TgAb), antimicrosomal antibody (TMAb)], tumor-associated status (tumor size, number and region of positive lymph nodes, and metastasis (TNM) stage), and thyroid disorders (presence or absence of HT, Graves disease, follicular adenoma, or malignant lymphoma) were obtained from a review of the medical records from the Patient Information Inquiry System. The normal ranges of serum fT3, fT4, TSH, Tg, TgAb, and TMAb were 3.67 to 10.43 pmol/L, 7.5 to 21.1 pmol/L, 0.35 to 4.94 mIU/L, 4.14 to 14.46 ng/ml, 0.0% to 30.0%, and 0.0% to 20.0%, respectively. We had free thyroid hormones, TSH, Tg, TgAb, and TMAb measured simultaneously with surgery. However, the measurement results of patients known to have taken thyroid medication were excluded. This research was approved by the Ethics Committee of the First Affiliated Hospital of Wenzhou Medical College.

The final diagnostic outcome was defined as the presence or absence of thyroid cancer. Data were analyzed using Statistical Package for Social Science (SPSS) version 13.0. The odds ratio (OR) in multivariate logistic regression analysis, with the relative 95% CI was calculated to assess the relevance of thyroid-associated clinical or biochemical conditions to predict the final diagnostic outcome. Confounding factors, including age, gender, and thyroid disorders, were investigated statistically. Comparisons of frequency distributions and numerical variable data were performed using the chi-square (χ^2^) test. Observed differences were considered statistically significant if the probability of chance occurrence was less than 0.05.

## Results

The clinical and biochemical features of patients with and without thyroid cancer were compared. Of the 1,024 patients with thyroid cancer, female patients (n = 843) accounted for the majority (female/male ratio = 4.66/1.00). The age distribution (range 10 to 84 years, mean 45.0 ± SD 12.1 years) revealed a predominance in the 30 to 44-year-old age group (n = 449 patients, 43.8%) with thyroid cancer. However, the high tumor incidence in the 45 to 60-year-old age group (n = 370 patients, 36.1%) should not be neglected. Comparison between the two groups highlighted a statistically significant difference in gender (χ^2^ = 5.990, *P* = 0.014), age (χ^2^ = 13.588, *P* = 0.018) (Table 1). To avoid the interference of several extreme values, fT3, fT4, TSH, Tg, TgAb, and TMAb were treated as categorical variables in the analysis. Significant differences were detected when the following values were compared: TSH (χ^2^ = 20.160, *P* <0.001), TgAb (χ^2^ = 14.142, *P* <0.001) and TMAb (χ^2^ = 15.026, *P* <0.001) but not fT3 (χ^2^ = 4.784, *P* = 0.306), fT4 (χ^2^ = 4.548, *P* = 0.337), and Tg (χ^2^ = 3.431, *P* = 0.180) among patients with and without thyroid cancer (Table 2). Moreover, the presence of HT was associated with thyroid cancer (χ^2^ = 67.421, *P* <0.001). Regarding calcification of the thyroid nodules, the incidence of microcalcification was higher in patients with thyroid cancer than in the controls (71.4% vs 16.2%, χ^2^ = 633.792, *P* <0.001). However, the prevalence of thyroid cancer was similar in patients with solitary nodules and patients with multiple nodules (χ^2^ = 1.456, *P* = 0.228) (Table 1).

**Table 1:** Comparison of characteristics of patients with and without thyroid cancer

**Characteristics**	**Thyroid cancer**		***P*-value**^**a**^
	**Present (n = 1,024)**	**Absent (n = 1,028)**	
Gender			0.014*
Male	181 (17.7)	226 (22.0)	
Female	843 (82.3)	802 (78.0)	
Age, yr			0.018*
0 ~ 14	3 (0.3)	5 (0.5)	
15~29	85 (8.3)	89 (8.7)	
30~44	449 (43.8)	382 (37.2)	
45~59	370 (36.1)	411 (40.0)	
60~74	103 (10.1)	133 (12.9)	
75~89	14 (1.4)	8 (0.8)	
Nodular type			0.228
Diffuse/multinodular goiter	651 (63.6)	627 (61.0)	
Solitary nodules	373 (36.4)	401 (39.0)	
Calcification			
Coarse calcifications			0.727
Absent	886 (86.5)	884 (86.0)	
Present	138 (13.5)	144 (14.0)	
Microcalcification			<0.001^*^
Absent	293 (28.6)	861 (83.8)	
Present	731 (71.4)	167 (16.2)	
HT			<0.001^*^
Absent	836 (81.6)	962 (93.6)	
Present	188 (18.4)	66 (6.4)	

Results are presented as number (%) of patients. ^a^Calculated using chi-square test; ^*^statistically significant (*P* <0.05). HT, Hashimoto’s thyroiditis.

**Table 2:** Comparison of the biochemical features of patients with and without thyroid cancer

**Characteristics**	**Thyroid cancer**		***P*-value**^**a**^
	**Present (n = 1,024)**	**Absent (n = 1,028)**	
The level of thyroid-associated hormone			
fT3, pmol/L^b^			0.306
<3.67	82 (9.2)	66 (7.3)	
3.67 to 4.42	263 (29.6)	287 (31.7)	
4.43 to 4.97	284 (32.0)	266 (29.4)	
4.98 to 10.43	258 (29.1)	283 (31.3)	
>10.43	1 (0.1)	2 (0.2)	
fT4, pmol/L^b^			0.337
<7.5	11 (1.2)	5 (0.6)	
7.5 to 12.83	301 (33.9)	282 (31.2)	
12.84 to 14.85	281 (31.6)	303 (33.5)	
14.86 to 21.1	283 (31.9)	299 (33.0)	
>21.1	12 (1.4)	16 (1.8)	
TSH, mIU/L^b^			<0.001^*^
<0.35	58 (6.1)	84 (8.7)	
0.35 to 1.17	258 (27.1)	312 (32.4)	
1.18 to 1.96	273 (28.6)	283 (29.4)	
1.97 to 4.94	317 (33.3)	247 (25.7)	
>4.94	47 (4.9)	36 (3.7)	
Tg, ng/ml			0.180
<4.14	131 (34.3)	107 (36.1)	
4.14 to 14.46	158 (41.4)	103 (34.8)	
>14.46	93 (24.3)	86 (29.1)	
Antibody^c^ (n = 748)			
TgAb			<0.001^*^
Negative	309 (78.4)	314 (88.7)	
Positive	85 (21.6)	40 (11.3)	
TMAb			<0.001^*^
Negative	309 (78.4)	315 (89.0)	
Positive	85 (21.6)	39 (11.0)	

Results are presented as number (%) of patients. ^a^Calculated using chi-square test; ^b^patients with free T3 (fT3), fT4, or thyroid-stimulating hormone (TSH) measurements within the normal range were divided into three tertiles of similar size, respectively; ^c^only 748 patients who had information on thyroglobulin antibody (TGAb) and antimicrosomal antibody (TMAb) were included in the analysis; ^*^statistically significant (*P* <0.05). Tg, thyroglobulin.

Subsequently, a binary logistic regression analysis was performed to define the risk predictors for thyroid cancer, which simultaneously analyzed gender, age, serum fT3, fT4 and TSH concentration, HT, and calcification. In addition, considering the feedback inhibition regulation of TSH by serum fT3 and fT4 concentrations, the interaction of variables between TSH and/or fT3, and/or fT4 were also taken into account in the analysis. Finally, four variables, namely, calcification, HT, TSH, and age, were entered into the multivariate model via forward stepwise regression (likelihood ratio). An increased risk of thyroid cancer was detected among the patients with HT (OR = 3.732, 95%CI = 2.563–5.435), microcalcification (OR = 14.486, 95%CI = 11.374–18.449). There was significantly increased risk of malignancy in patients with serum TSH of 1.97 to 4.94 mIU/L, compared to patients with TSH below 0.35 mIU/L (OR = 1.951, 95% CI = 1.201–3.171, *P* = 0.007) (Table 3).

**Table 3:** Independent risk predictors for diagnosis of thyroid malignancy under multivariate logistic regression analysis (n = 1,789 patients)

**Variable**	**Adjusted odds ratio**	**95% CI**	***P*-value**
Age, yr			0.004^*^
0 ~ 14	1.00		
15~29	1.217	0.164, 9.032	0.848
30~44	1.544	0.215, 11.092	0.666
45~59	1.188	0.165, 8.550	0.864
60~74	0.724	0.098, 5.321	0.751
75~89	2.852	0.300, 27.138	0.362
TSH, mIU/L^a^			0.001^*^
<0.35	1.00		
0.35 to 1.17	1.079	0.663, 1.756	0.759
1.18 to 1.96	1.357	0.835, 2.207	0.218
1.97 to 4.94	1.951	1.201, 3.171	0.007^*^
>4.94	1.235	0.598, 2.550	0.568
HT			
Absent	1.00		
Present	3.732	2.563, 5.435	<0.001^*^
Calcification			
Coarsecalcifications	1.00		
Microcalcification	14.486	11.374, 18.449	<0.001^*^

^a^Patients with free T3 (fT3), fT4, or TSH measurements within the normal range were divided into tertiles of similar size, respectively. Variables assigned odds ratios of 1.0 were used as the reference for multivariate analysis. ^*^Statistically significant (*P* <0.05).

The patients suffering from PTC (n = 1004, 98.05%) were selected and divided into patients with HT (group I) and patients without HT (group II) based on the final histologic examination. Tables 4 and 5 show the clinical and pathologic features of the two groups; 18.63% of the patients with PTC had concurrent HT, which was more frequent than in the benign group (*P* <0.001). Moreover, the female patients constituted an overwhelming 97.3% (n = 182) of the patients with coexisting PTC and HT. The mean age at initial thyroidectomy was similar between the groups (χ^2^ = 7.298, *P* = 0.063), as well as thyroid-associated disorders (χ^2^ = 3.322, *P* = 0.325) (Table 4). Furthermore, the tumor size (χ^2^ = 2.975, *P* = 0.209), frequency of occult PTC (χ^2^ = 2.872, *P* = 0.090), extrathyroidal invasion (χ^2^ = 0.885, *P* = 0.347), capsular infiltration (χ^2^ = 0.891, *P* = 0.345), angioinvasion (χ^2^ = 0.429, *P* = 0.512), and lymph node metastases (χ^2^ = 0.227, *P* = 0.634) did not differ between patients with and without HT. No distant metastasis was observed in the two groups. Although the majority of positive lymph nodes (58.3%) in patients with HT were distributed in level VI (classification references [21]) and a higher proportion was observed in group I with stage I (85.6%), the difference was not significant compared with group II (χ^2^ = 4.703, *P* = 0.427; χ^2^ = 0.540, *P* = 0.910, respectively), in other words, the pathologic TNM (pTNM) classification in the two groups were similar (shown in Table 5).

**Table 4:** Clinical features of patients with papillary thyroid cancer by group in patients with and without Hashimoto’s thyroiditis (HT)

**Characteristics**	**Group I**	**Group II**	**χ**^**2**^	***P*-value**
	**(with HT)**	**(without HT)**		
	**n = 187**	**n = 817**		
Age, yr			7.298	0.063
<30	23 (12.3)	65 (8.0)		
30~44	76 (40.6)	362 (44.3)		
45~59	74 (39.6)	291 (35.6)		
>60	14 (7.5)	99 (12.1)		
Gender			35.082	<0.001^*^
Male	5 (2.7)	171 (20.9)		
Female	182 (97.3)	646 (79.1)		
Thyroid-associated disorders			3.322	0.325
Nodular goiter	56 (81.2)	258 (82.2)		
Follicular adenoma	8 (11.6)	45 (14.3)		
Graves disease	4 (5.8)	10 (3.2)		
Malignant lymphoma	1 (1.4)	1 (0.3)		

Results are presented as number (%) of patients. ^*^Statistically significant (P < 0.05).

**Table 5:** Pathologic features of patients with papillary thyroid cancer (PTC) by group in patients with and without Hashimoto’s thyroiditis (HT)

**Characteristics**	**Group I**	**Group II**	**χ**^**2**^	***P*-value**
	**(with HT)**	**(without HT)**		
	**n = 187**	**n = 817**		
Pathological TNM staging			0.540	0.910
I	160 (85.6)	687 (84.1)		
II	4 (2.1)	25 (3.1)		
III	15 (8.0)	70 (8.6)		
IV	8 (4.3)	35 (4.3)		
Tumor size			2.975	0.209
≤1 cm	126 (67.4)	496 (60.7)		
1 to 4 cm	59 (31.6)	304 (37.2)		
>4 cm	2 (1.1)	17 (2.1)		
Frequency of occult PTC			2.872	0.090
Absent	61 (32.6)	321(39.3)		
Present	126 (67.4)	496 (60.7)		
Lymph nodes metastases			0.227	0.634
Absent	129 (69.0)	578 (70.7)		
Present	58 (31.0)	239 (29.3)		
Distribution of positive lymph nodes			4.703	0.427
Level VI	49 (58.3)	217 (54.5)		
Level II	9 (10.7)	33 (8.3)		
Level III	9 (10.7)	48 (12.1)		
Level IV	10 (11.9)	47 (11.8)		
Level V	6 (7.1)	52 (13.1)		
Level I	1 (1.2)	1 (0.3)		
Extrathyroidal invasion			0.885	0.347
Absent	184 (98.4)	794 (97.2)		
Present	3 (1.6)	23 (2.8)		
Capsular infiltration			0.891	0.345
Absent	159 (85.0)	671 (82.1)		
Present	28 (15.0)	146 (17.9)		
Vascular invasion			0.429	0.512
Absent	186 (99.5)	815 (99.8)		
Present	1 (0.5)	2 (0.2)		
Distant metastases	0	0		

Results for Groups I and II are presented as number (%) of patients. ^*^Statistically significant (*P* <0.05).

## Discussion

As shown in the current study, the peak incidence of thyroid cancer in Wenzhou occurs in the 30 to 44-year-old age group. Moreover, when the association between gender and thyroid cancer was assessed, significantly increased rates of malignancy were detected among the female patients (82.3%) (χ^2^ = 5.990, *P* = 0.014). However, further logistic regression analysis did not identify gender as an independent risk predictor. These results may challenge a previously recognized view of an increased risk of underlying malignancies in male patients [5,9,22,23]. Furthermore, the patient age was demonstrated as a risk predictor for malignancy. Increased adjusted ORs for thyroid cancer were observed in the older age group (75 years) in accordance with previous reports [22,24], although no significant difference was observed compared with the young age group (<15 years, *P* = 0.362).

The risk of thyroid cancer has been shown to increase with serum TSH concentrations, and even within normal ranges, higher TSH levels are associated with a higher incidence and more advanced stage of thyroid malignant tumor [9,10,24,25]. Serum TSH has a tropic effect on thyroid nodules and suppression of TSH concentration by administration of exogenous thyroxine may interfere with the growth of established nodules, as well as the formation of new thyroid nodules [26-29]. TSH suppression is also associated with a decreased frequency of PTC [30].

We demonstrated that the risk of thyroid cancer increased in parallel with the serum TSH concentration within the normal range at presentation. This observation is supported by a previous study [24]. In our study, serum TSH concentration (>4.94 mIU/L) was not associated with the greatest risk of thyroid cancer, which is inconsistent with other studies [8,9]. We have no vigorous explanation for this inconsistency. One reason may be that the sample size of the group with serum TSH concentrations >4.94 mIU/L was smaller than the other groups. In addition, the mechanism of TSH on thyroid disease may differ between populations. Furthermore, several arguments weaken the role of TSH in the development or progression of malignant thyroid tumors: 1) TSH receptor mutations in regions functionally associated with increased signal transduction do not often occur in malignant thyroid tumors [31]; 2) other growth factors such as insulin-like growth factor-I (IGF-I) have been demonstrated to have a more important role in the growth of thyroid cancer in *in vitro* studies [32,33], and TSH needs cooperation with insulin/IGF-1 to educe its proliferative effects [34]; 3) Shi *et al*. [35] reported that the relationship between TSH receptor mRNA levels and the aggressiveness of cancer was inversed; 4) thyroid cancer occurs in patients with serum TSH concentrations suppressed by hyperfunctioning nodules in the contralateral lobe [36]; 5) a recent study showed that patients carrying one of two alleles associated with an increased risk of differentiated thyroid cancer have lower serum TSH concentrations [37].

Taken together, TSH is unlikely to act versatilely in cancer development. These findings indicate that serum TSH concentration can be used as an adjunct biochemical predictor of thyroid cancer. However, this requires further investigation.

In contrast to previous studies [7,38] that reported that Tg levels were significantly higher in patients with thyroid cancer than in those with benign disorders, no significant difference in Tg concentration was observed in patients with malignancy. Limited to the current measurement, whether preoperative serum Tg measurement helps differentiate benign from malignant thyroid disorders needs further verification. Ultrasonography cannot reliably distinguish benign from malignant lesions [39]. The current study demonstrates similar incidences of malignancy between patients with solitary nodules and those with diffuse or multinodular lesions, as reported by ultrasonography, although others found that the presence of solitary nodules found by palpation is associated with increased malignancy rates [5,9,40,41]. In addition, a higher risk of malignancy was detected in patients with microcalcification (OR = 14.486, *P* <0.001, Table 3), which is consistent with recent investigations in patients evaluated through ultrasonography [42,43]. Nevertheless, approximately 13.5% of patients with thyroid cancer (n = 138) were confirmed to have coexisting coarse calcifications. Accordingly, ultrasonographic findings, such as solitary nodules or multinodular types, as well as microcalcifications or coarse calcifications, should not prevent further evaluation of benign or malignant lesions.

In the present study, a vast majority of thyroid malignancies were PTC (98.05%) and HT was found to coincide highly with PTC (18.63%). Moreover, further regression analysis identified the presence of HT as an independent risk predictor for malignancy (OR = 3.732, *P* <0.001, Table 3). These data confirm the large American dataset from Dr. Skahen’s report of 1955 [12]. Subsequent studies showed a similar association between PTC and HT. Although according to previous reports, hypothyroidism induced by HT could induce high serum TSH concentration, which may enhance the proliferative activity of follicular epithelia and result in the development of PTC [44], the oncogenic effect that causes PTC in patients with HT are still unknown. According to Harach *et al*. [45], iodine intake may play a role in the tumorigenesis of thyroiditis. Although Wenzhou is an iodine-sufficient area, determining the correlation between HT and PTC in terms of iodine intake is difficult because no information on iodine intake was available in the majority of patients studied. Some molecular mechanisms, such as the PI3K/Akt or RET/RAS/ERK pathways may also contribute to tumorigenesis [20,46]. At present, no evidence suggests that HT is a premalignant lesion for PTC. Furthermore, in contrast to previous studies that reported that the presence of HT in PTC is associated with better prognosis, lower recurrence, and a less aggressive disorder than cases without HT [47-49], the current results did not find that HT has a protective effect on tumor aggressiveness in patients with PTC, which is consistent with the recent reports by Del Rio *et al*. [50]. Recently, Kim *et al*. reported that HT was positively associated with multifocality and smaller size but not with extrathyroidal invasion, nodal metastasis, or TNM stage [51]. These divergences may be due, at least in part, to differences in patient selection (this retrospective study only included patients undergoing thyroidectomy, thus, a percentage of patients with HT that are treated conservatively may have been omitted), or to indications for thyroidectomy, inclusion of different histologic types (others included PTC as well as FTC), or varying definitions of thyroiditis. Nevertheless, the current results indicate that the presence of HT represents an increased risk of developing thyroid cancer, particularly PTC, but may have a minimum effect on the aggressiveness of the malignancy.

## Conclusions

In conclusion, the risk of thyroid malignancy increases with the presence of HT and microcalcification as evaluated by ultrasonography. Raised TSH levels within the normal range are also independently associated with the likelihood of thyroid malignancy. Coexisting HT in PTC does not have a significant effect on the biologic behavior of PTC. Based on the current findings, because the study was retrospective, we do not advocate a more aggressive role for surgical intervention for patients with HT, microcalcification, or even high TSH levels. Prospective large-scale studies are required to verify or establish the potentially important factors for predicting thyroid malignancy, and the follow-up of the patients with HT in PTC should be done to verify long-term prognosis in clinical practice.

## Consent

Written informed consent was obtained from the patient for publication of this report and any accompanying images.

## Abbreviations

HT: Hashimoto’s thyroiditis; PTC: Papillary thyroid cancer; TSH: Thyrotropin; TSH: Thyroid stimulating hormone; Tg: Thyroglobulin; FNA: Fine-needle aspiration; FTC: Follicular thyroid cancer; fT4: free T4; fT3: free T3; TgAb: Thyroglobulin antibody; TMAb: antimicrosomal antibody; OR: Odds ratio.

## Competing interests

The authors declare that they have no competing interests.

## Authors’ contributions

Z-QY made contributions to the acquisition and analysis of clinical data and drafted the manuscript. D-NG participated in the design of the study and performed the statistical analysis. H-YH and Y-LZ conceived of the study, and participated in its design and coordination and helped to draft the manuscript. X-QH drafted the manuscript. X-HZ critically revised the manuscript for important intellectual content and gave final approval of the version to be published. All authors read and approved the final manuscript.

## References

[B1] Sherman SI Thyroid carcinoma Lancet 2003 361 501 511 10.1016/S0140-6736(03)12488-9 12583960

[B2] Zheng W Zhang Z An analysis of cancer incidence in 2005 in Lucheng District, Wenzhou City, Zhejiang Province Bulletin of Chinese Cancer 2007 16 306 308

[B3] Marqusee E Benson CB Frates MC Doubilet PM Larsen PR Cibas ES Mandel SJ Usefulness of ultrasonography in the management of nodular thyroid disease Ann Intern Med 2000 133 696 700 10.7326/0003-4819-133-9-200011070-00011 11074902

[B4] Shibata Y Yamashita S Masyakin VB Panasyuk GD Nagataki S 15 years after Chernobyl: new evidence of thyroid cancer Lancet 2001 358 1965 1966 10.1016/S0140-6736(01)06971-9 11747925

[B5] Hegedus L Bonnema SJ Bennedbaek FN Management of simple nodular goiter: current status and future perspectives Endocr Rev 2003 24 102 132 10.1210/er.2002-0016 12588812

[B6] Yeung MJ Serpell JW Management of the solitary thyroid nodule Oncologist 2008 13 105 112 10.1634/theoncologist.2007-0212 18305054

[B7] Christensen SB Bondeson L Ericsson UB Lindholm K Prediction of malignancy in the solitary thyroid nodule by physical examination, thyroid scan, fine-needle biopsy and serum thyroglobulin. A prospective study of 100 surgically treated patients Acta Chir Scand 1984 150 433 439 6495973

[B8] Stocker DJ Burch HB Thyroid cancer yield in patients with Graves' disease Minerva Endocrinol 2003 28 205 212 14605602

[B9] Boelaert K Horacek J Holder RL Watkinson JC Sheppard MC Franklyn JA Serum thyrotropin concentration as a novel predictor of malignancy in thyroid nodules investigated by fine-needle aspiration J Clin Endocrinol Metab 2006 91 4295 4301 10.1210/jc.2006-0527 16868053

[B10] Haymart MR Repplinger DJ Leverson GE Elson DF Sippel RS Jaume JC Chen H Higher serum thyroid stimulating hormone level in thyroid nodule patients is associated with greater risks of differentiated thyroid cancer and advanced tumor stage J Clin Endocrinol Metab 2008 93 809 814 18160464 10.1210/jc.2007-2215 PMC2266959

[B11] Cipolla C Sandonato L Graceffa G Fricano S Torcivia A Vieni S Latteri S Latteri MA Hashimoto thyroiditis coexistent with papillary thyroid carcinoma Am Surg 2005 71 874 878 16468540

[B12] Dailey ME Lindsay S Skahen R Relation of thyroid neoplasms to Hashimoto disease of the thyroid gland AMA 1955 70 291 297 10.1001/archsurg.1955.01270080137023 13227748

[B13] Crile G Jr Struma lymphomatosa and carcinoma of the thyroid Surg Gynecol Obstet 1978 147 350 352 581102

[B14] Crile G Jr Hazard JB Incidence of cancer in struma lymphomatosa Surg Gynecol Obstet 1962 115 101 103 13882216

[B15] Holm LE Blomgren H Lowhagen T Cancer risks in patients with chronic lymphocytic thyroiditis N Eng J Med 1985 312 601 604 10.1056/NEJM198503073121001 3838363

[B16] Ott RA McCall AR McHenry C Jarosz H Armin A Lawrence AM Paloyan E The incidence of thyroid carcinoma in Hashimoto's thyroiditis Am Surg 1987 53 442 445 3605864

[B17] Shands WC Carcinoma of the thyroid in association with struma lymphomatosa (Hashimoto's disease) Ann Surg 1960 151 675 682 10.1097/00000658-196005000-00008 14445445 PMC1613703

[B18] Singh B Shaha AR Trivedi H Carew JF Poluri A Shah JP Coexistent Hashimoto's thyroiditis with papillary thyroid carcinoma: impact on presentation, management, and outcome Surgery 1999 126 1070 1076 discussion 1076–1077 10.1067/msy.2099.101431 10598190

[B19] Fiore E Rago T Latrofa F Provenzale MA Piaggi P Delitala A Scutari M Basolo F Di Coscio G Grasso L Hashimoto's thyroiditis is associated with papillary thyroid carcinoma: role of TSH and of treatment with L-thyroxine Endocr Relat Cancer 2011 18 429 437 10.1530/ERC-11-0028 21565972

[B20] Larson SD Jackson LN Riall TS Uchida T Thomas RP Qiu S Evers BM Increased incidence of well-differentiated thyroid cancer associated with Hashimoto thyroiditis and the role of the PI3k/Akt pathway J Am Coll Surg 2007 204 764 773 discussion 773–765 10.1016/j.jamcollsurg.2006.12.037 17481480 PMC2430882

[B21] Robbins KT Shaha AR Medina JE Califano JA Wolf GT Ferlito A Som PM Day TA Consensus statement on the classification and terminology of neck dissection Arch Otolaryngol Head Neck Surg 2008 134 536 538 10.1001/archotol.134.5.536 18490577

[B22] Hegedus L Clinical practice. The thyroid nodule N Eng J Med 2004 351 1764 1771 10.1056/NEJMcp031436 15496625

[B23] Mazzaferri EL Management of a solitary thyroid nodule N Eng J Med 1993 328 553 559 10.1056/NEJM199302253280807 8426623

[B24] Polyzos SA Kita M Efstathiadou Z Poulakos P Slavakis A Sofianou D Flaris N Leontsini M Kourtis A Avramidis A Serum thyrotropin concentration as a biochemical predictor of thyroid malignancy in patients presenting with thyroid nodules J Cancer Res Clin Oncol 2008 134 953 960 10.1007/s00432-008-0373-7 18327610 PMC12160766

[B25] Jonklaas J Nsouli-Maktabi H Soldin SJ Endogenous thyrotropin and triiodothyronine concentrations in individuals with thyroid cancer Thyroid 2008 18 943 952 10.1089/thy.2008.0061 18788918 PMC2879493

[B26] Gharib H Changing trends in thyroid practice: understanding nodular thyroid disease Endocr Pract 2004 10 31 39 15251619 10.4158/EP.10.1.31

[B27] Papini E Petrucci L Guglielmi R Panunzi C Rinaldi R Bacci V Crescenzi A Nardi F Fabbrini R Pacella CM Long-term changes in nodular goiter: a 5-year prospective randomized trial of levothyroxine suppressive therapy for benign cold thyroid nodules J Clin Endocrinol Metab 1998 83 780 783 10.1210/jc.83.3.780 9506726

[B28] Vermiglio F Lo Presti VP Violi MA Moleti M Castagna MG Finocchiaro MD Mattina F Mandolfino M Zimbaro G Trimarchi F Changes in both size and cytological features of thyroid nodule after levothyroxine treatment Clin Endocrinol 2003 59 347 353 10.1046/j.1365-2265.2003.01854.x 12919158

[B29] Zelmanovitz F Genro S Gross JL Suppressive therapy with levothyroxine for solitary thyroid nodules: a double-blind controlled clinical study and cumulative meta-analyses J Clin Endocrinol Metab 1998 83 3881 3885 10.1210/jc.83.11.3881 9814462

[B30] Fiore E Rago T Provenzale MA Scutari M Ugolini C Basolo F Di Coscio G Miccoli P Grasso L Pinchera A Vitti P L-thyroxine-treated patients with nodular goiter have lower serum TSH and lower frequency of papillary thyroid cancer: results of a cross-sectional study on 27 914 patients Endocr Relat Cancer 2010 17 231 239 10.1677/ERC-09-0251 20167722

[B31] Matsuo K Friedman E Gejman PV Fagin JA The thyrotropin receptor (TSH-R) is not an oncogene for thyroid tumors: structural studies of the TSH-R and the alpha-subunit of Gs in human thyroid neoplasms J Clin Endocrinol Metab 1993 76 1446 1451 10.1210/jc.76.6.1446 8501149

[B32] Derwahl M Broecker M Kraiem Z Clinical review 101: Thyrotropin may not be the dominant growth factor in benign and malignant thyroid tumors J Clin Endocrinol Metab 1999 84 829 834 10.1210/jc.84.3.829 10084556

[B33] Mazzaferri EL Thyroid cancer and Graves' disease: the controversy ten years later Endocr Pract 2000 6 221 225 10.4158/EP.6.2.221 11421537

[B34] Kimura T Van Keymeulen A Golstein J Fusco A Dumont JE Roger PP Regulation of thyroid cell proliferation by TSH and other factors: a critical evaluation of in vitro models Endocr Rev 2001 22 631 656 10.1210/er.22.5.631 11588145

[B35] Shi Y Zou M Farid NR Expression of thyrotrophin receptor gene in thyroid carcinoma is associated with a good prognosis Clin Endocrinol 1993 39 269 274 10.1111/j.1365-2265.1993.tb02365.x 8222289

[B36] Satta MA De Rosa G Testa A Maussier ML Valenza V Rabitti C Saletnich I D'Ugo D Picciocchi A Thyroid cancer in suppressed contralateral lobe of patients with hot thyroid nodule Eur J Cancer 1993 29A 1190 1192 8518032 10.1016/s0959-8049(05)80313-2

[B37] Gudmundsson J Sulem P Gudbjartsson DF Jonasson JG Sigurdsson A Bergthorsson JT He H Blondal T Geller F Jakobsdottir M Common variants on 9q22.33 and 14q13.3 predispose to thyroid cancer in European populations Nat Genet 2009 41 460 464 10.1038/ng.339 19198613 PMC3664837

[B38] Spencer CA Serum thyroglobulin measurements: clinical utility and technical limitations in the management of patients with differentiated thyroid carcinomas Endocr Pract 2000 6 481 484 11155225

[B39] Hegedus L Thyroid ultrasound Endocrinol Metab Clin North Am 2001 30 339 360 viii-ix 10.1016/S0889-8529(05)70190-0 11444166

[B40] Franklyn JA Daykin J Young J Oates GD Sheppard MC Fine needle aspiration cytology in diffuse or multinodular goitre compared with solitary thyroid nodules BMJ 1993 307 240 10.1136/bmj.307.6898.240 8369686 PMC1678158

[B41] Mazzaferri EL Santos ET Dl Rofagha-Keyhani S Solitary thyroid nodule: diagnosis and management MCNA 1988 72 1177 1211 10.1016/s0025-7125(16)30736-2 3045454

[B42] Frates MC Benson CB Doubilet PM Kunreuther E Contreras M Cibas ES Orcutt J Moore FD Jr Larsen PR Marqusee E Alexander EK Prevalence and distribution of carcinoma in patients with solitary and multiple thyroid nodules on sonography J Clin Endocrinol Metab 2006 91 3411 3417 10.1210/jc.2006-0690 16835280

[B43] Wang N Xu Y Ge C Guo R Guo K Association of sonographically detected calcification with thyroid carcinoma Head Neck 2006 28 1077 1083 10.1002/hed.20481 17022090

[B44] Okayasu I Fujiwara M Hara Y Tanaka Y Rose NR Association of chronic lymphocytic thyroiditis and thyroid papillary carcinoma. A study of surgical cases among Japanese, and white and African Americans Cancer 1995 76 2312 2318 10.1002/1097-0142(19951201)76:11<2312::AID-CNCR2820761120>3.0.CO;2-H 8635037

[B45] Harach HR Ceballos GA Thyroid cancer, thyroiditis and dietary iodine: a review based on the Salta, Argentina model Endocr Pathol 2008 19 209 220 10.1007/s12022-008-9038-y 18696273

[B46] Kang DY Kim KH Kim JM Kim SH Kim JY Baik HW Kim YS High prevalence of RET, RAS, and ERK expression in Hashimoto's thyroiditis and in papillary thyroid carcinoma in the Korean population Thyroid 2007 17 1031 1038 10.1089/thy.2007.0035 17900235

[B47] Kashima K Yokoyama S Noguchi S Murakami N Yamashita H Watanabe S Uchino S Toda M Sasaki A Daa T Nakayama I Chronic thyroiditis as a favorable prognostic factor in papillary thyroid carcinoma Thyroid 1998 8 197 202 10.1089/thy.1998.8.197 9545105

[B48] Loh KC Greenspan FS Dong F Miller TR Yeo PP Influence of lymphocytic thyroiditis on the prognostic outcome of patients with papillary thyroid carcinoma J Clin Endocrinol Metab 1999 84 458 463 10.1210/jc.84.2.458 10022401

[B49] Matsubayashi S Kawai K Matsumoto Y Mukuta T Morita T Hirai K Matsuzuka F Kakudoh K Kuma K Tamai H The correlation between papillary thyroid carcinoma and lymphocytic infiltration in the thyroid gland J Clin Endocrinol Metab 1995 80 3421 3424 10.1210/jc.80.12.3421 8530576

[B50] Del Rio P Cataldo S Sommaruga L Concione L Arcuri MF Sianesi M The association between papillary carcinoma and chronic lymphocytic thyroiditis: does it modify the prognosis of cancer? Minerva Endocrinol 2008 33 1 5 18277374

[B51] Kim KW Park YJ Kim EH Park SY Park Do J Ahn SH Jang HC Cho BY Elevated risk of papillary thyroid cancer in Korean patients with Hashimoto's thyroiditis Head Neck 2011 33 691 695 10.1002/hed.21518 21484918

